# *Describ*ePS*P* and *ProPSP*: German Multicenter Networks for Standardized Prospective Collection of Clinical Data, Imaging Data, and Biomaterials of Patients With Progressive Supranuclear Palsy

**DOI:** 10.3389/fneur.2021.644064

**Published:** 2021-05-25

**Authors:** Gesine Respondek, Günter U. Höglinger

**Affiliations:** ^1^Department of Neurology, Hannover Medical School, Hanover, Germany; ^2^German Center for Neurodegenerative Diseases, Munich, Germany; ^3^Department of Neurology, Technical University of Munich, Munich, Germany

**Keywords:** disease networks, progressive supranuclear palsy, corticobasal syndrome, rare neurological disease, natural history, biobank

## Abstract

**Background:** The German research networks *DescribePSP* and *ProPSP* prospectively collect comprehensive clinical data, imaging data and biomaterials of patients with a clinical diagnosis of progressive supranuclear palsy. Progressive supranuclear palsy is a rare, adult-onset, neurodegenerative disease with striking clinical heterogeneity. Since now, prospective natural history data are largely lacking. Clinical research into treatment strategies has been limited due to delay in clinical diagnosis and lack of natural history data on distinct clinical phenotypes.

**Methods:** The *DescribePSP* network is organized by the German Center for Neurodegenerative Diseases. *DescribePSP* is embedded in a larger network with parallel cohorts of other neurodegenerative diseases and healthy controls. The *DescribePSP* network is directly linked to other *Describe* cohorts with other primary diagnoses of the neurodegenerative and vascular disease spectrums and also to an autopsy program for clinico-pathological correlation. The *ProPSP* network is organized by the German Parkinson and Movement Disorders Society. Both networks follow the same core protocol for patient recruitment and collection of data, imaging and biomaterials. Both networks host a web-based data registry and a central biorepository. Inclusion/exclusion criteria follow the 2017 Movement Disorder Society criteria for the clinical diagnosis of progressive supranuclear palsy.

**Results:** Both networks started recruitment of patients by the end of 2015. As of November 2020, *N* = 354 and 269 patients were recruited into the *DescribePSP* and the *ProPSP* studies, respectively, and *N* = 131 and 87 patients received at least one follow-up visit.

**Conclusions:** The *DescribePSP* and *ProPSP* networks are ideal resources for comprehensive natural history data of PSP, including imaging data and biological samples. In contrast to previous natural history studies, *DescribePSP* and *ProPSP* include not only patients with Richardson's syndrome, but also variant PSP phenotypes as well as patients at very early disease stages, before a diagnosis of possible or probable PSP can be made. This will allow for identification and evaluation of early biomarkers for diagnosis, prognosis, and progression.

## Introduction

In 2015, *two* German multicenter research networks, *DescribePSP* and *ProPSP*, were set up by the authors with the ultimate goal to improve early clinical diagnosis, monitoring, and prediction of disease progression in patients with progressive supranuclear palsy (PSP).

*DescribePSP* and *ProPSP* are acronyms. DEsCRIbE stands for “DZNE Clinical Register Study of Neurodegenerative Disorders.” DescribePSP is the register study for PSP patients. ProPSP stands for “Prospective observational study to investigate demography, clinical course and biomarkers of PSP.”

PSP is a rare neurodegenerative disease, defined by the unique neuropathology, which is characterized by intracellular aggregation of the microtubule-associated protein tau (1). Onset of first symptoms occurs usually between the 5th and the 7th decade and mean disease duration is approximately 8 years (2, 3). Clinico-pathological studies suggest that PSP has previously been underdiagnosed during lifetime and that the correct ante-mortem diagnosis of PSP has been delayed for several years, due to a lack of specific symptoms at early disease stages and due to heterogeneous clinical presentations (2, 4). Variant clinical phenotypes of PSP (vPSP) have been described in multiple clinico-pathological studies, which differ from the classical Richardson's syndrome not only with regard to the initial clinical manifestation, but also with regard to progression rate and survival (5). Former clinical diagnostic criteria for PSP, the National Institute of Neurological Disorders and Stroke and the Society for PSP criteria [NINDS-SPSP criteria (6)] preferentially recognized patients with Richardson's syndrome, and therefore lacked sensitivity for the broader spectrum of PSP manifestations (7). Although treatment strategies are presently restricted to symptomatic therapies, several tau targeting therapies are being developed for PSP (1). These developments further increase the need for correct and early clinical diagnosis of PSP and reliable prediction of disease progression, to set the stage for early disease-modifying interventions.

To reduce diagnostic delay and to improve diagnostic sensitivity, the new Movement Disorder Society clinical diagnostic criteria for PSP, short MDS-PSP criteria, introduced the diagnostic category “suggestive of PSP” (s.o. PSP) alongside with “possible PSP” and “probable PSP” (8). S.o. PSP represents the lowest level of diagnostic certainty and significantly increases diagnostic sensitivity and reduces time to diagnosis for PSP according to retrospective studies with autopsy cases (9–11). However, the sensitivity, specificity, positive and negative predictive value of the diagnosis of s.o. PSP has not been studied prospectively so far.

The main goals of the *DescribePSP* and the *ProPSP* networks are to collect prospective natural history data of patients with PSP, to prospectively validate the new MDS-PSP criteria, and ultimately to improve early clinical diagnosis, monitoring, and prediction of disease progression in patients with PSP. These two networks collaborate synergistically and were set up separately mainly for organizational reasons.

In this paper, we outline the *DescribePSP* and the *ProPSP* network structures as well as study designs and achievements of both networks up to now.

## Methods

*DescribePSP* and *ProPSP* share many similarities with regard to methodology, including criteria for patient inclusion and collection of clinical data, imaging data and biomaterials. However, there are some organizational and methodological differences between both cohort studies. For a better overview, differences between *DescribePSP* and *ProPSP* are also summarized in Table 1.

**Table 1:** Methodological differences between *DescribePSP* and *ProPSP*.

	***DescribePSP***	***ProPSP***
Organization	German Center for Neurodegenerative Diseases (DZNE)	German Parkinson's Association (DPG)
Recruitment centers	Affiliated with the DZNE (Figure 1)	Affiliated with the DPG (Figure 1)
Web-based database	WebSpirit	MACRO, Elsevier®
Central imaging platform	XNAT	Not provided
Follow-up schedule	12-months follow-ups	6-months follow-ups
Parallel cohorts	Other neurodegenerative diseases and healthy controls	Not provided
Brain banking	Central brain banking program	Individual neuropathological institutes

### Network Structures

*DescribePSP* is organized by German Center for Neurodegenerative Diseases (DZNE), which is a member of the Helmholtz Association and is funded by the German Federal Ministry of Education and Research (BMBF) and the German federal states (Bundesländer) in which DZNE sites are located. The steering committee of the *DescribePSP* network consists of the principal investigator (G. Höglinger, Deputy G. Respondek) and a representative of the database management, as well as a principle investigator representative per recruitment center.

**Figure 1:**
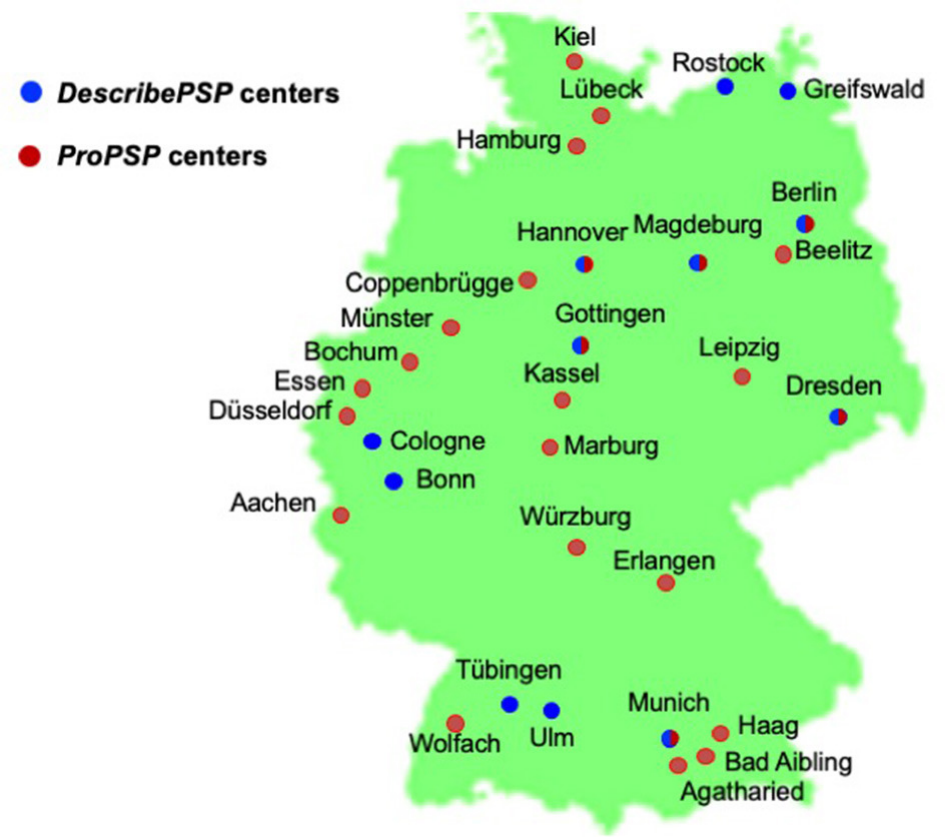
Geographic distribution of *DescribePSP* and *ProPSP* centers throughout Germany.

**Figure 2:**
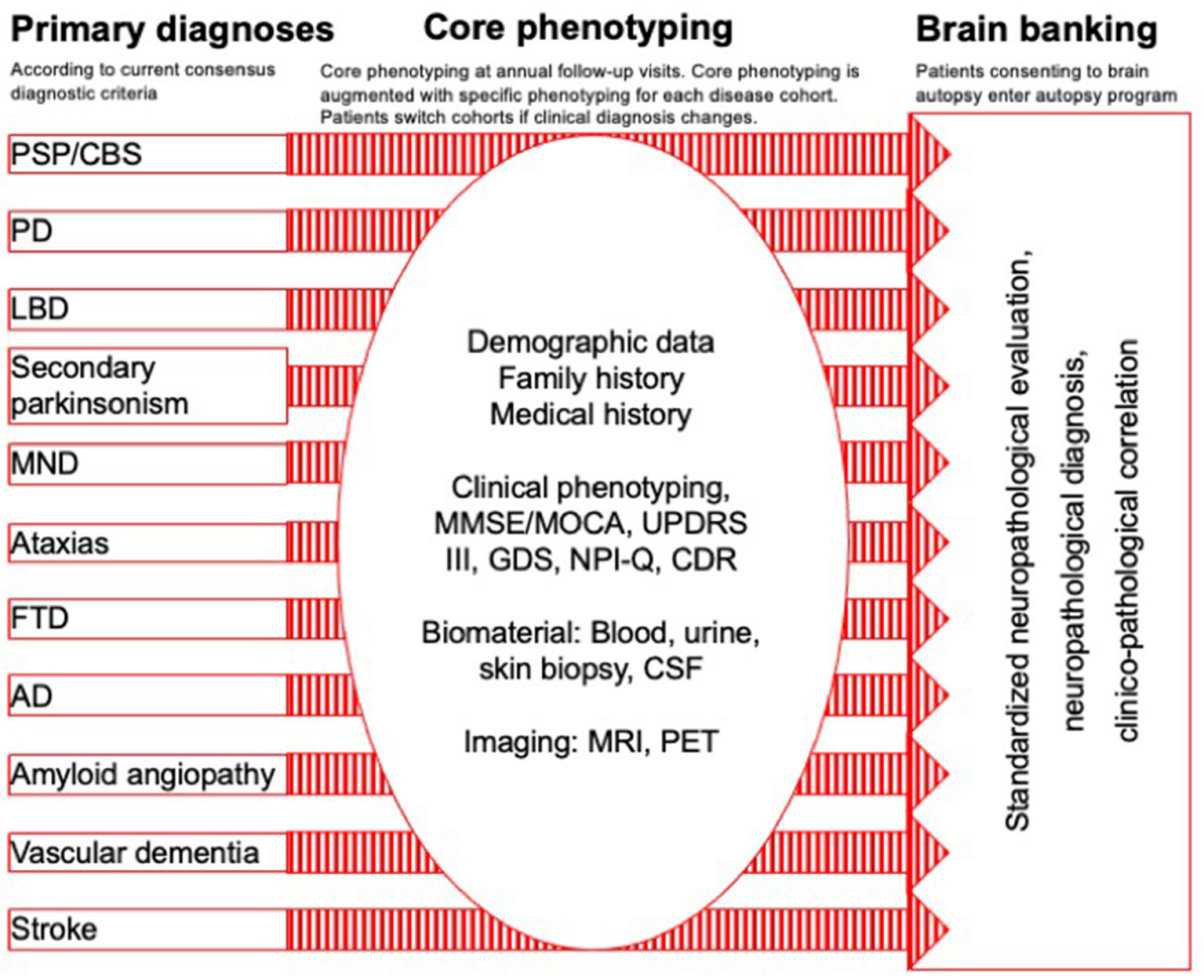
Describe parallel cohort design: primary diagnoses, core phenotyping, and brain banking.

*DescribePSP* recruitment centers currently comprise of 11 tertiary care centers with expertise in movement disorders and other neurodegenerative diseases, which are located in Berlin, Bonn, Dresden, Gottingen, Greifswald, Hanover, Cologne, Magdeburg, Munich, Rostock, and Tubingen (Figure 1). The central data management and the central biorepository of *DescribePSP* are located at the DZNE headquarters in Bonn. The *DescribePSP* study is embedded in a larger network with parallel *Describe* cohorts that recruit other neurodegenerative diseases, including Alzheimer's disease (AD), frontotemporal dementia (FTD), Parkinson's disease (PD), motoneuron disease (MND), ataxias, and vascular diseases, including stroke, cerebral amyloid angiopathy, as well as healthy controls (Figure 2).

*ProPSP* is organized within the German Parkinson and Movement Disorders Society (https://www.parkinson-gesellschaft.de), which is a non-profit organization based in Berlin. The *ProPSP* network is also supported by the German PSP Association (https://www.psp-gesellschaft.de), which is a patient support group and a non-profit organization.

The steering committee of the *ProPSP* network consists of the principal investigator (G. Höglinger, Deputy G. Respondek), a representative of the database management, and a representative of the recruitment centers elected by a simple majority.

The *ProPSP* study currently comprises of 25 centers with expertise in movement disorders and other neurodegenerative diseases, which are located in Aachen, Agatharied, Bad Aibling, Beelitz, Berlin, Bochum, Coppenbrugge, Dresden, Dusseldorf, Erlangen, Essen, Haag, Hamburg, Hanover, Kassel, Leipzig, Lubeck, Magdeburg, Marburg, Munich, Munster, Rostock, Ulm, Wolfach, and Wurzburg (Figure 1).

The study sites Berlin, Dresden, Gottingen, Hanover, Magdeburg, and Munich have access to both networks (Figure 1) and recruit patients randomly either into the *DescribePSP* or into the *ProPSP* study.

### Study Design

*DescribePSP* and *ProPSP* are both multicenter longitudinal observational studies for PSP in Germany. Both networks prospectively follow up patients with a clinical diagnosis of PSP and collect comprehensive longitudinal natural history data, imaging and biomaterials according to the same core protocol.

Each network runs a central web-based data registry and a central biorepository.

#### Inclusion Criteria

Since 2017, inclusion criteria for both studies are the MDS-PSP diagnostic criteria (8). As defined by the MDS-PSP diagnostic criteria, patients with corticobasal syndrome (CBS) receive a diagnosis of s.o. or possible PSP with predominant CBS (PSP-CBS) (8) and are therefore also recruited into both cohorts.

At the time of the initiation of both studies in 2015 and until 2017, inclusion criteria for both studies were the NINDS-SPSP criteria (6). Patients that meet the NINDS-SPSP criteria also meet the MDS-PSP diagnostic criteria.

#### Recruitment of Participants

Participants are consecutively recruited into both studies through referrals from the associated outpatient or inpatient clinic of the recruitment centers. Patients who meet the inclusion criteria and give written informed consent are enrolled.

#### Follow-up Schedule

The follow-up intervals are set to 6 months in the *ProPSP* study and to 12 months in the *DescribePSP* study. If the patient or the recruitment center cannot comply with this schedule, smaller or larger follow-up intervals are permitted without specific restrictions.

#### Termination Criteria

The observation period of the individual participant ends in both studies with the withdrawal of the participant's consent, with the death of the patients, or with the termination of study. Patients can withdraw their consent at any time and without stating reasons. They can request anonymization or deletion of their stored data. This only applies if the data has not already been released to other researchers or anonymized. A medical decision can also be made to terminate the study if the continuation of the study would result in an unjustifiable burden for the patient or if the patient does not fulfill inclusion criteria anymore.

#### Acquisition of Clinical Data, Biomaterial, and Imaging Data

Both, the *DescribePSP* and the *ProPSP* networks follow the same core protocol with regard to acquisition of clinical data, biomaterial, and imaging as shown in Table 2. At baseline visit, demographic data, medical history, medication, family history, and education and job history are collected and are updated at every follow-up visit. The diagnostic certainty level as well as the PSP predominance type according to the MDS-PSP diagnostic criteria (8) are documented at every visit.

**Table 2:** *DescribePSP* and *ProPSP* core protocol.

**Inclusion criteria**	**MDS-PSP diagnostic criteria for probable, possible, and suggestive of PSP (8)**
Clinical phenotyping	PSP-specific clinical scales: • PSP Rating Scale (PSPRS) (12) • PSP Staging System (PSP-SS) (13) • PSP-Quality of Life Scale (PSP-QoL) (14) • PSP-Clinical Deficits Scale (PSP-CDS) (15)Parkinsonism-specific clinical scales: • Schwab and England Disability Scale (SEADL) (16) • MDS-Unified Parkinson's Disease Rating Scale (MDS-UPDRS) Part III (17) • Starkstein Apathy Scale (SAS) (18)Generic clinical scales: • Clinical Global Impression—Severity Scale (CGI-s) (19) • Geriatric Depression Scale: a 30 item a self-report assessment used to assess current mood in elderly patientes (20) • Montreal Cognitive Assessment (MoCA) (21)
Biobanking	Blood, RNA/DNA, CSF, urine, skin biopsy
Imaging	MRI: MPRAGE, DTI, SWI, T2, FLAIR
Brain banking	Histopathological evaluation

#### Storage of Clinical Data and Imaging Data

Each recruitment center enters the collected data into an electronic case report form (CRF) on a central, web-based data platform. For *DescribePSP*, the central data platform is managed by the DZNE headquarter in Bonn. The *DescribePSP* data platform uses the clinical data-management system WebSpirit. The *DescribePSP* network uses XNAT for a separate imaging platform.

For *ProPSP*, the central data platform is provided by the “Münchner Studienzentrum” (https://www.mri.tum.de/studienzentrum) at the Klinikum rechts der Isar, Technical University of Munich in Munich. The *ProPSP* data platform uses the software MACRO Electronic Data Capture by Elsevier®. The *ProPSP* does not run a separate imaging platform to upload MR images, but collects information in the central data platform on date and place of the MRI, MR sequences and atrophy patterns.

#### Storage of Biomaterial

The biomaterials collected within the *DescribePSP* network are centrally stored in the biorepository of the DZNE in Bonn. For *ProPSP*, central storage is currently reorganized and will be transferred from the Technical University of Munich to the Hannover Unified Biobank at Hanover Medical School in Hanover.

#### Brain Banking

During the participation in *DescribePSP* and *ProPSP*, the patients and their caregivers are informed about the option of post mortem brain autopsy for verification of the clinical diagnosis and brain banking for research purposes. Written informed consent is obtained by the clinician involved in the patient's care.

*DescribePSP* and all other *Describe* cohorts have a central brain banking program run by the DZNE (https://www.dzne.de/en/research/brain-bank/) with the neuropathological institute located in Tubingen, Germany. It allows for central clinico-pathological correlation and verification of the clinical diagnosis, if the patient consented to autopsy. For *ProPSP*, brain banking is performed in individual neuropathological institutes that are collaborating with the *ProPSP* recruitment centers and is therefore not centralized. If patients within the *ProPSP* study consent in post-mortem brain autopsy, they are also asked to consent in the correlation of their collected clinical data and the histopathological data generated by the respective neuropathological institute.

## Results

Since initiation of both networks, extensive natural history data, imaging data and biomaterials of patients with a clinical diagnosis of s.o. PSP, possible PSP, and probable PSP according to the MDS-PSP criteria have been collected within both, the *DescribePSP* and the *ProPSP* networks.

The following preliminary results are available for *DescribePSP* and *ProPSP* as of November 2020.

A total of 354 patients with a clinical diagnosis of PSP have been enrolled into *DescribePSP*, and 131 patients have completed at least one follow-up visit. A total of 269 patients with a clinical diagnosis of PSP have been enrolled into *ProPSP* (Table 3), and 87 patients have completed at least one follow-up visit. Preliminary patient characteristics of *DescribePSP* and *ProPSP* at baseline are shown in Table 3.

**Table 3:** Baseline characteristics of patients.

	**DescribePSP**	**ProPSP**
Number of recruited patients (as of November 2020)	354	269
Age in years (mean ± SD [range])	71.6 ± 7.7 [46–87]	69.8 ± 6.9 [51–85]
Disease duration in months (mean ± SD [range])	60 ± 36 [14–222]	51 ± 34 [1–189]
Gender (male in %)	60.3	54.8
PSPRS total score (mean ± SD [range])	34 ± 12.8 [10–75]	35 ± 14.4 [3–76]
PSP-SS (mean ± SD [range])	3 ± 1.1 [1–5]	3 ± 1.1 [1–5]
PSP-CDS total score (mean ± SD [range])	6 ± 2.4 [1–13]	8 ± 3.1 [2–18]

*PSPRS, PSP Rating Scale (12); PSP-SS, PSP Staging System (13); PSP-CDS, PSP-Clinical Deficits Scale (15)*.

Biological samples from 298 *DescribePSP* participants, including blood, RNA, DNA, CSF, urine, and skin biopsies have been collected, and standardized MR imaging from 85 *DescribePSP* participants has been performed and uploaded to the *DescribePSP* imaging platform. As of November 2020, five patients from the *DescribePSP* study entered the brain bank program.

Approximately 25% of participants in both, the *DescribePSP* and the *ProPSP* studies did not complete follow-up according to schedule. Reasons for termination included (1) deceased, (2) patient's or caregiver's wish, (3) lost to follow-up, (4) immobility, (5) participation in interventional trial, and (6) moved away.

Clinical data, imaging and biomaterials from both networks have been shared with national and international collaborators for projects that serve the primary goal of *DescribePSP* and *ProPSP*. The Progressive Supranuclear Palsy Clinical Deficits Scale (PSP-CDS), a clinical scale to monitor clinical deficits in patients with PSP across its broad phenotypes, has been developed with baseline and follow-up clinical datasets of *DescribePSP* (exploratory) and *ProPSP* (confirmatory) (15).

For the creation of a modified version of the Progressive Supranuclear Ratings Scale [PSPRS (12)], longitudinal datasets from *DescribePSP, ProPSP*, and from the TAUROS trial (22) have been analyzed (23).

*DescribePSP* and *ProPSP* have served as platforms to recruit patients for a video tutorial that demonstrates diagnostic symptoms of different PSP phenotypes (24). Novel tau PET tracers for PSP were established at two *DescribePSP* centers (Cologne, Munich) (25, 26). Patients of the *DescribePSP* and *ProPSP* cohorts received 18F-GE-180 PET imaging which detected microglial activation in the brain of patients with PSP and CBS (27). A subset of patients from the *DescribePSP* and *ProPSP* cohorts has entered into a genetic study that demonstrated genetic determinants of survival in PSP (28).

*DescribePSP* and *ProPSP* have served as trial ready cohorts to recruit patients with PSP into interventional trials (29, 30).

## Discussion

*DescribePSP* and *ProPSP* are unique and synergistic research networks in Germany to prospectively study the natural history of patients with PSP.

Both networks comprise of centers with specialization in movement disorders and other degenerative diseases. Although the organizational structure of both networks differs, they follow the same core protocol with regard to inclusion criteria and collection of clinical data, imaging data and biomaterials, which allows for high quality comparisons between both cohorts.

There are some organizational differences between both networks. *DescribePSP* has a parallel cohort design, which allows for good comparison of collected data and biomaterials between different primary diagnoses. *DescribePSP* has central brain banking, while brain banking in *ProPSP* is decentralized at the moment. *ProPSP* has a higher number of recruitment centers, which results from the fact that centers that are not affiliated to the DZNE can also participate. *ProPSP* uses follow-up intervals of 6 months instead of 12 months, which might increase the probability of collecting follow-up data in patients that would not return after 12 months due to severe immobility. However, the utility of this shorter follow-up interval still needs to be evaluated.

In contrast to previous natural history studies in PSP [for review: (4)], which included only patients with clinical presentation of Richardson's syndrome, *DescribePSP* and *ProPSP* networks recruit patients with diagnoses of s.o. PSP and vPSP according to the MDS-PSP criteria (8). S.o. PSP was designed to serve for early identification of individuals who may develop “possible PSP” or “probable PSP” as the disease evolves, “thereby justifying close clinical follow-up examinations, especially in longitudinal observational studies to further characterize the natural history of PSP with the overall goal of improving diagnosis of patients in early-stage disease”(8).

**Figure 3:**
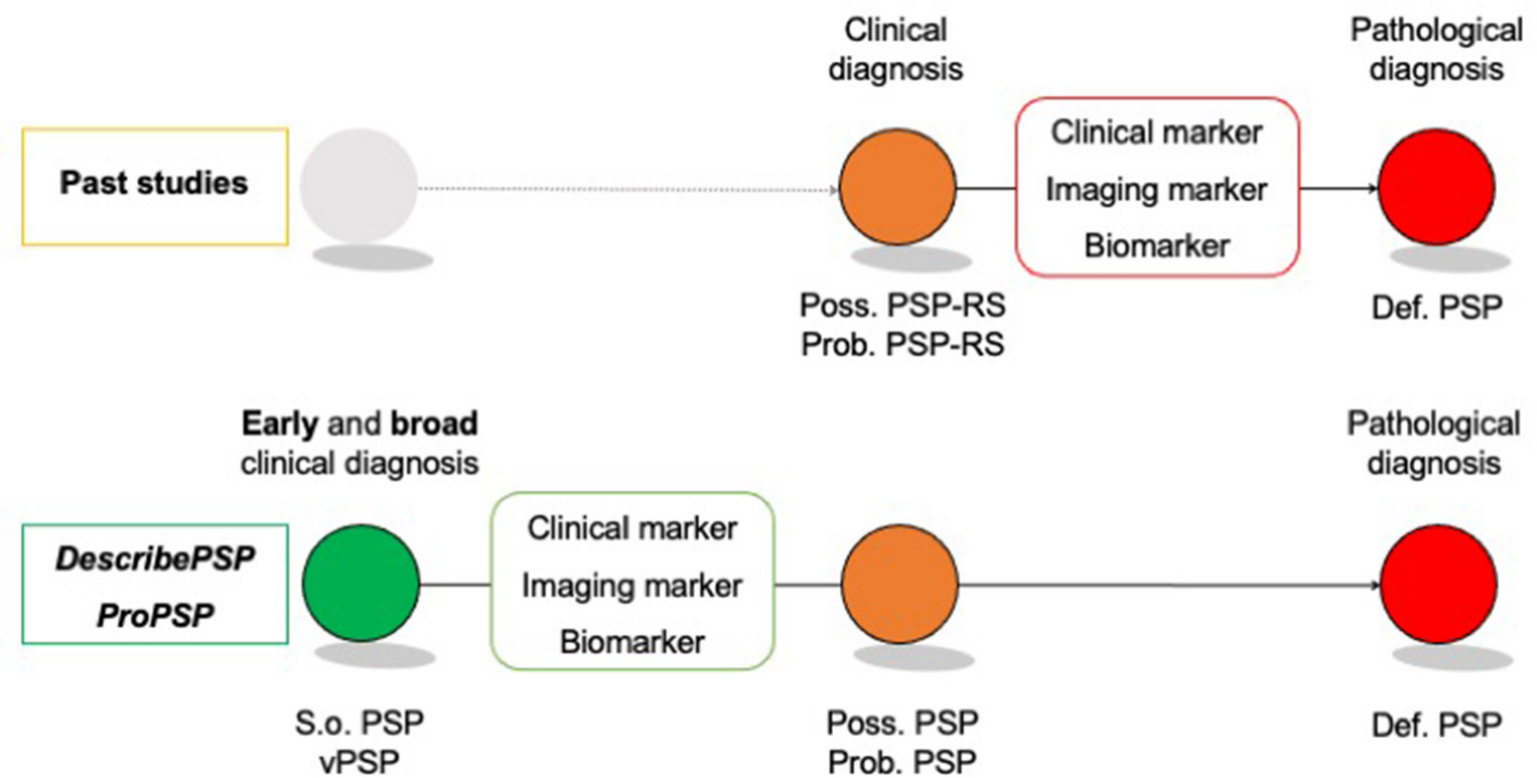
Identification of early disease markers in “suggestive” of and “variant PSP”. Def. PSP, Definite PSP; Poss. PSP, Possible PSP; Prob. PSP, Probable PSP; S.o. PSP, Suggestive of PSP; vPSP, Variant PSP.

The *DescribePSP* and the *ProPSP* cohorts will serve as invaluable resources to study the specificity of s.o. PSP and vPSP for underlying PSP pathology and to allow for identification and evaluation of early biomarkers for diagnosis, prognosis, and progression (Figure 3).

## Data Availability Statement

The original contributions presented in the study are included in the article/supplementary material, further inquiries can be directed to the corresponding author/s.

## Ethics Statement

The studies involving human participants were reviewed and approved by the Ethic committees of all centers involved in the presented studies. The patients/participants provided their written informed consent to participate in this study.

## Author Contributions

All authors listed have made a substantial, direct, and intellectual contribution to the work and approved it for publication.

## Conflict of Interest

The authors declare that the research was conducted in the absence of any commercial or financial relationships that could be construed as a potential conflict of interest.

## Appendices

**Appendix 1. The *DESCRIBE-PSP* study group:**

Thomas Gasser, Andreas Hermann, Günter Höglinger, Matthias Höllerhage, Okka Kimmich, Thomas Klockgether, Andrea Kühn, Johannes Levin, Gerrit Machetanz, Antje Osterrath, Carla Palleis, Johannes Prudlo, Gesine Respondek, and Annika Spottke.

**Appendix 2. The ProPSP study group:**

Daniela Berg, Katrin Bürk, Joseph Claßen, Georg Ebersbach, Carsten Eggers, Imke Galazky, Lennard Herrmann, Günter Höglinger, Matthias Höllerhage, Klaus Jahn, Wolfgang Jost, Jan Kassubek, Martin Klietz, Andrea Kühn, Franz Marxreiter, Steffen Paschen, Elmar Pinkhardt, Monika Pötter-Nerger, Gesine Respondek, Johannes Schwarz, Lars Tönges, Claudia Trenkwalder, Tobias Warnecke, Florian Wegner, and Jürgen Winkler.
